# Serotonin transporter downregulation is associated with aortic stenosis, and early profibrotic remodeling is mitigated by pharmacological inhibition of HTR2B receptor

**DOI:** 10.3389/fcvm.2026.1729078

**Published:** 2026-02-12

**Authors:** Dov Levine, Chiara Camillo, Estibaliz Castillero, Emre Bektik, Yaagnik Kosuri, Anthony Campbell, Cary Karcher, Abba Krieger, Liming Pei, Ting Peng, Nicole Zeak, Erfan Faridmoayer, Mark A. Oyama, Juan B. Grau, Robert J. Levy, Giovanni Ferrari

**Affiliations:** 1 Irving Medical Center, Department of Surgery, Columbia University, New York, NY, United States; 2 Department of Statistics and Data Science, Wharton School, University of Pennsylvania, Philadelphia, PA, United States; 3 Department of Pathology and Laboratory Medicine, Perelman School of Medicine, University of Pennsylvania, Philadelphia, PA, United States; 4 Department of Cardiothoracic Surgery, The Valley Hospital Heart Institute, Ridgewood, NJ, United States; 5 Pediatric Heart Valve Center and the Division of Cardiology, Children’s Hospital of Philadelphia, Philadelphia, PA, United States

**Keywords:** aortic stenosis, valvular interstitial cell, serotonin transporter, serotonin receptor 2B, angiotensin II

## Abstract

**Background:**

Aortic Stenosis (AS) is a highly prevalent disease involving physiological and structural remodeling of aortic valve, yet lacks effective medical therapy to halt its progression. Serotonin (5HT) signaling has been implicated in valvular disease. We hypothesized that AS is associated with impaired 5HT clearance due to reduced serotonin receptor (SERT) expression and increased 5HT receptor (HTR) activity.

**Methods:**

Sixty-six patients with severe AS undergoing aortic valve (AV) replacement were enrolled in the study, and samples from their explanted AV were harvested. Anatomically normal control AVs were obtained from transplant donors. Explanted AVs were collected for gene expression analysis and 5-HTTLPR genotyping. Gene expression was assessed by RT2-profiler gene array. *In vivo*, 8-week-old mice received 28-day Angiotensin-II (AngII) infusions ± HTR2B antagonist (LY272015) through subcutaneous Alzet osmotic-pump implants. AV structure and function were assessed via echocardiography, histology, and RNA sequencing. Human aortic valve interstitial cells (AVICs) were treated with AngII ± SERT siRNAs to assess 5HT signaling and profibrotic/procalcific markers.

**Results:**

AS patients exhibited reduced SERT and increased HTR signaling. AngII ± SERT siRNA promoted VIC osteogenic marker expression. In mice, AngII caused AV thickening, increased velocities and gradients, and activation of fibrosis and mildly calcification-related gene sets, including serotonin, TGF*β*, Wnt/*β*-catenin, PI3K/Akt, and Notch pathways. Pharmacological inhibition of HTR2B preserved AV structure, normalized transvalvular velocities and pressure gradients, and reversed AngII-induced transcriptional changes.

**Conclusions:**

In human and mouse AVs, reduced SERT expression and increased HTR2B signaling contribute to early-onset fibro-calcific remodeling. HTR2B inhibition by LY272015 reverses these effects, suggesting it as a potential therapeutic strategy for fibrotic remodeling in AS.

## Introduction

Calcific aortic valve disease (CAVD) refers to a spectrum of disorders starting with structural remodeling of the valve without hemodynamic compromise, or aortic sclerosis, and culminating in outflow obstruction, or aortic stenosis (AS) (1). Due to population growth and an aging population, the rates of CAVD are increasing, with a global prevalence of 12.6 million people (2). Thirty percent of people above 65 have aortic valve sclerosis, and of these, 10% progress to symptomatic AS within 10 years (1). Effective pharmacological treatments for aortic valve disease remain limited, underscoring the need for a deeper understanding of underlying cellular and molecular mechanisms driving disease progression.

Serotonin, or 5-hydroxytryptamine, (5HT) is a monoamine neurotransmitter, with various neurologic and systemic effects (3). The 5HT receptor (HTR) subtype most critical for cardiovascular physiology, HTR2B, promotes intracellular calcium influx, nitric oxide synthase activity, cellular proliferation, and secretion of inflammatory cytokines and extracellular matrix (ECM) components (3). SERT is a transmembrane protein that processes 5HT intracellularly, thereby limiting HTR activity and maintaining homeostasis (4). 5HT is implicated in heart valve disease, observed in carcinoid syndrome or in association with medications, such as the diet drug, Dexfenfluramine, a 5HT transporter (SERT) inhibitor and HTR2B agonist (5, 6). *SERT* is subject to a polymorphism in the promoter (non-expressed) region of the gene (7); this polymorphism, abbreviated 5-HTTLPR, is a variable number tandem repeat (VNTR), 43 bases in length. Multiple studies have attempted to link 5-HTTLPR genotypes to serotonin-related disorders. We recently reported that *SERT* promoter polymorphism 5-HTTLPR LL genotype is associated with mitral valve (MV) surgery at younger age in patients with degenerative mitral regurgitation (7). To date, it is still not known to what extent 5-HTTLPR genotypes affect SERT activity in AV disease. It has been reported that 5HT signaling is linked with Angiotensin-II (AngII), with a resulting alteration of valve interstitial cell (VICs) contractility and remodeling (8). HTR2B receptors work in concert with the AngII type 1 receptor (AT1R) to mediate hypertrophic signaling in cardiac fibroblasts (9). Finally, AngII and the renin-angiotensin system (RAS) have been reported to contribute to numerous cardiac and valvular diseases (4).

At the cellular level, AV stenosis is marked by extracellular matrix (ECM) remodeling, calcification with valvular interstitial cell activation toward an osteogenic-like phenotype, and oxidative stress (10–12). Prior research by our group regarding 5HT mechanisms has previously focused on MV disease, where increased HTR2B and decreased SERT are observed in degenerative mitral regurgitation (7, 13). In our current study, we hypothesized that 5HT dysfunction impacts the progression of AV stenosis due to AngII. To test this hypothesis, we focused on two clinically relevant scenarios: (1) HTR2B activation and (2) decreased SERT, utilizing both murine and human models of AV disease. Our results confirm that impaired 5HT metabolism exacerbates the pathological valvular response to AngII, and that blocking related HTR2B signaling can mitigate AV disease progression.

## Methods

### Study design

The rationale and objective of this study was to determine the impact of altered 5HT and AngII signaling in the development and progression of AV disease. Clinical data, AV specimens from patients with AS and normal AV samples were collected and analyzed. Identification of 5-HTTLPR genotype was performed using DNA samples isolated from patients' blood samples with IRB approval. All mice studies were performed with IACUC approval. C57BL/6J wild type mice treated with or without AngII and/or LY272015 were employed to investigate the effect of AngII signaling and HTR2B blockade in AV remodeling. Power analyses were conducted to determine minimum sample sizes, with an alpha (*p*-value) of 0.5 to achieve a power of 0.8.

### Mice anesthesia and euthanasia

For survival surgeries, mice were anesthetized using isoflurane (induction at 3%–4% and maintenance at 1.5%–2% in an oxygenated chamber) delivered via a precision vaporizer. Depth of anesthesia was confirmed by the absence of pedal withdrawal reflex and regular respiratory rate before any manipulation. Body temperature was maintained using a thermostatically controlled heating pad throughout the procedure.

For non-survival surgeries, anesthesia methods were applied same as above prior to euthanasia. Under deep anesthesia, mice were positioned supine on a heated surgical platform to maintain body temperature. Then, thoracotomy was performed, and the hearts were perfused *in situ* via right and left atrium with PBS to remove blood from circulation. After perfusion, the hearts were excised and collected for downstream analyses. As these were non-survival procedures, animals did not regain consciousness and were euthanized by exsanguination and confirmed by cervical dislocation at the conclusion of tissue collection. All personnel performing these procedures were trained and certified in rodent anesthesia and euthanasia techniques.

### AngII chronic infusion model

All experiments on mice were approved by the IACUC at Columbia University (Protocol# AABS7622) prior to the study. 8-week-old wild type male mice, Jackson Lab, (C57BL/6J) were fed a hypercholesterolemic diet (Breslow Western Diet, Fisher and Son Inc. Cat#: TD1810724) and infused with AngII (1,000 ng/kg/min) using osmotic pumps (Alzet 2004) for 28 days, as previously described (12, 13). Osmotic pumps were implanted subcutaneously on the back, posterior to scapulae, through a small skin incision. LY272015 treatment (3 mg/kg in sterile PBS) was given twice/week over the 28-day AngII infusion by intraperitoneal injection, as previously described (13). After 28 days, a subset of mice in the AngII and AngII+LY groups had their pumps removed and survived for an additional 48 h. They were then anesthetized with isoflurane, followed by heart perfusion with PBS and sample collection. Heart tissue was collected for subsequent experiments. Group sizes were 12–15 mice per group in those not receiving AngII (control and LY272015), and 18–20 mice per group in those receiving AngII (AngII alone and AngII+LY), to account for an expected 10% mortality from aortic aneurysm/rupture and due to planned pump removal (*n* = 7–8 per group). One mouse from each AngII and AngII+LY died, the former on day 30, after pump removal, and latter on day 20 due to abdominal aortic aneurysm/rupture. A subset of hearts (*n* = 5/group) were stored in formalin for subsequent histological analysis. A separate subset of hearts (*n* ≥ 5/group) were micro-dissected and aortic valve leaflets were processed for bulk RNA sequencing.

### Murine blood pressure measurement

Blood pressure measurements were performed in conscious mice using a computerized tail cuff method (Coda 4.2.2; Kent Scientific Corporation, Torrington, CT, USA). Mice were placed in an animal holder on a warming platform until the tail temperature was verified between 32 and 35 °C. Blood pressure results were averaged over ten repeated measurements for each mouse, after five acclimation cycles, as previously described (14). Data were collected at the culmination of 28 days and 24 h after pump removal.

### Murine echocardiography

After both 28 days of AngII infusion, and 48 h after pump removal, B-mode ultrasound with color Doppler using the right parasternal long-axis view (RPLAX) was additionally performed to assess aortic valve (AV) function under isoflurane anesthesia (15, 16). AV velocity was measured using pulsed-wave Doppler imaging and included peak and mean systolic velocity and pressure gradients. M-mode ultrasound using parasternal short-axis view (PSAX) was additionally performed to assess LV function (15, 16). Characterization of left ventricular outflow tract (LVOT), aortic valve (AV), ascending aorta (AA) was performed. Echocardiography and data analyses done using Fujifilm Visualsonics VEVO 3100 High Frequency Ultrasound Imaging System and VEVO Lab software, respectively, by experienced sonographers who were blinded during the study, as previously described (14).

### Human specimens

All human subjects research in this study, including the use of human tissues, conformed to the principles outlined in the Declaration of Helsinki. Patients with AS referred for first time surgery at the participating hospitals from 2009 to 2022 were enrolled in this study. Informed consent per IRB approval was obtained at either The Hospital of the University of Pennsylvania (IRB Protocol #809349), The Valley Hospital (IRB Protocol#11.0009), or Columbia University Irving Medical Center (IRB Protocol #AAAR6796) upon admission prior to surgery. All patient information was de-identified. Diseased aortic valves due to AS were collected after surgical explant. Exclusion criteria for this study included Marfan's syndrome, congenital valve abnormalities, endocarditis, rheumatic heart disease, history of cancer, autoimmune diseases, previous aortic surgery, and any history of cardiac trauma. SSRI use prior to surgery was ascertained by searching for the following drugs: Citalopram, Fluoxetine, Fluvoxamine, Paroxetine, Sertraline, and Vilazodone. Control AV tissue was isolated from healthy hearts from cardiac donors that were allocated for cardiac transplant but ultimately not transplanted for logistic reasons. Subjects with no known cardiopulmonary disease whose organs were listed but were unable to be placed at the time of organ recovery for heart transplantation, and who consented to donate tissue for research purposes by LiveOn New York (previously New York Organ Donor Network), were included in this study.

### 5-HTTLPR genotyping

Identification of 5-HTTLPR genotype was performed in DNA isolated from buffy coat from blood, when available, or myocardium tissue when blood was not available (normal hearts from group ii). DNA was isolated with a QIAamp DNA Mini Kit (Qiagen). The SERT promoter was amplified with specific primers (5-HTTLPR forward, 5′-TCCTCCGCTTTGGCGCCTCTTCC-3′ and 5-HTTLPR reverse, 5′-TGGGGGTTGCAGGGGAGATCCTG-3′). The fragment sizes were determined by agarose gel or determined by high throughput fragment analysis (3730 DNA Analyzer, GeneMate, Thermo Fisher Scientific) with results analyzed with PeakScannerTM2 software (Thermo Fisher Scientific).

### Gene expression by RT2 profiler

doiAV tissue for RNA analysis was flash frozen in liquid nitrogen. RNA samples suitable for RT2 PCR array profiler analyses were isolated using the RNeasy Fibrous Tissue Mini Kit (Qiagen). RNA concentrations were measured by Nanodrop technology (Thermo Fisher Scientific), and RNA integrity was assessed with the Agilent 2100 Bioanalyzer (Agilent). cDNA was prepared from RNA using the RT2 first strand system (Qiagen). qRT-PCR analyses were performed using RT2 Profiler Arrays (Qiagen) with specificity for dopamine-serotonin related gene expression. Each array contained a panel of proprietary controls to monitor genomic DNA contamination as well as the first strand synthesis, and real-time PCR efficiency (Qiagen). The “Dopamine-5HT” RT2 Profiler arrays contained 96 target genes, as well as housekeeping genes for normalization that included: Actin-beta, Beta-2-microglobulin, Glyceraldehyde-3-phosphate dehydrogenase, and Ribosomal protein-large-P0. Gene expression and fold changes were calculated using the 2^(−ΔΔCt)^ method.

### Human AVIC isolation and culture

Human AV leaflets were minced and digested with 0.25 mg/mL collagenase type 2 and 30 IU/mL hyaluronidase in complete DMEM for 16h–24 h at 37°C. Cryo Recovered VICs at passages 2–5 were used for all experiments. AVICs were treated for 7 days with 100 μM H_2_O_2_, 100 nM AngII or vehicle in complete growth medium, with treatment/medium replaced every 48 h. Replicates correspond to biological replicates.

### SERT silencing

Control AVICs were subjected to siRNA-mediated knockdown. AVICs were transfected with ON-TARGETplus SMARTpool siRNAs for SERT or nontargeting Ctrl pool (scrambled) (Dharmacon, Lafayette, CO) with the transfection reagent Lipofectamine RNAiMAX (Invitrogen, Grand Island, NY). The siRNA constructs were added to FBS-free OptiMEM medium at a concentration of 165nM in combination with lipofectamine. After 4 h, the medium was changed to fresh DMEM containing 10% FBS and incubated for 72 h. AVIC were subsequently treated for 7 days with 100 nM AngII or vehicle.

### Real time PCR for human VICs

Isolation of total mRNA for real-time PCR from human VICs was performed using the RNeasy mini kit (Qiagen). RNA concentration was measured on a DS11 spectrophotometer (DeNovix). 20–50 ng of RNA were retro-transcribed with a Maxima H Minus cDNA Synthesis Master Mix kit (Thermo Fisher Scientific), with equal amount of template RNA in each sample compared. Gene expression was measured with Thermo Fisher Scientific Taqman probes (Table 1) in a QuantStudio™ 5 Real-Time PCR System (Applied Biosystems, Thermo Fisher Scientific). GAPDH was used as a housekeeping gene.

**Table 1:** TaqMan probes for human gene expression analysis.

Gene	TaqMan assay ID
HTR2A	Hs06626790_s1
HTR2B	Hs00168362_m1
ACTA2	Hs00426835_g1
COL1A1	Hs00164004_m1
SPP1	Hs00959010_m1
RUNX2	Hs01047973_m1
SERT	Hs00984349_m1
TGFβ	Hs00998133_m1
Gapdh	Hs02786624_G1

### Histology and immunohistochemistry of AV tissue

Histology was performed in 5 µm sections from normal human AV leaflets and stenotic AV leaflets with the following stains/antibodies. Von Kossa (Polysciences 24633-1), and Alizarin Red (Sigma A5533) as markers of calcium deposition. Picrosirius red stain (Abcam AB150681); marker of fibrosis. Standard, single day staining protocols were carried out for Von Kossa, Alizarin red and Picrosirius red each including deparaffinization, hydration, stain incubation, counterstaining, dehydration and mounting steps. Care was taken to adjust pH of Alizarin red to 4.1–4.3 using 10% ammonium hydroxide. Additionally, direct UV light exposure was required for developing the Von Kossa brown staining in sections. A two-day immunohistochemistry protocol was used for HTR2B receptor (ProteinTech, 26408-1-AP). Paraffin-embedded leaflets were deparaffinized and hydrated with 100% xylene, 100% ethanol, 70% ethanol and deionized water washes. An antigen retrieval step using 1× citrate performed for immunohistochemistry of HTR2B. A blocking step performed on slides with 3% BSA in PBS following 5-minute wash with 1× DAKO. Sections incubated in primary antibodies dilution at 4°C overnight. Endogenous peroxidase was quenched in the following day using 3% H_2_O_2_. This was followed by incubation in secondary antibodies, DAB chromogen development and nuclear hematoxylin staining. Sections were dehydrated in reverse order using 70% ethanol, 100% ethanol, 100% xylene, then completed with permount mounting medium and glass cover slips. Assessment of images was performed in a blinded fashion using simple light microscopy under 5× and 10× magnification. The Molecular Pathology/MPSR Core (HICCC) at Columbia University was utilized for cutting sections from paraffin blocks as well as Hematoxylin and Eosin staining.

### Immunofluorescence of human aortic valves (SERT and HTR2B)

Human AVs were embedded in paraffin blocks and cut into 5 µm sections. Paraffin-embedded AV leaflets were deparaffinized and hydrated with successive 100% xylene, 100% ethanol, 70% ethanol and deionized water washes. The antigen retrieval step was performed using 1× citrate in a distilled water steamer for 30 min. Blocking was performed for 1 h at room temperature with 3% BSA-Tween (0.1%) in 1× PBS. Target proteins were stained within separate sections: SERT and HTR2B. Primary antibody for SERT (Thermo Fisher Scientific 711108) at 1:100 dilution was incubated at 4 °C overnight. Primary antibody for HTR2B (Invitrogen 720256) at 1:500 dilution was incubated at 4°C overnight. Alexa-Fluor-555 secondary antibodies (Thermo Fisher Scientific) at 1:400 incubated for 1 h at room temperature in addition to DAPI at 1:5000. Coverslips were mounted on slides with ProLong™ Diamond Antifade Mountant (Thermo Fisher Scientific). Sections were analyzed by using a Nikon Ti Eclipse inverted microscope with A1 scanning confocal microscope. 10x/0.37 and 20x/0.75 objectives were employed. 1,024 × 1,024 pixel images were acquired using NIS Elements software. The acquisition was performed by adopting laser power, gain, and offset settings that allowed maintaining pixel intensities within the 0–255 range to avoid saturation. Settings for each antibody were maintained among sessions. Assessment of images was performed in a blinded fashion.

### RNA sequencing

Bulk RNA-seq raw fast files were first subjected to quality control using FastQC (v0.12.1) to identify potential issues such as contamination and adapter sequences (Supplementary Figure S3). After trimming low-quality sequences, FastQC was rerun on the cleaned reads to ensure that all major quality issues were resolved. The sequencing reads were then aligned to the mouse reference genome (GRCm39) using HISAT2. Gene expression levels were quantified with feature Counts and differential expression analysis was conducted using DESeq2. The differentially expressed genes (DEGs) between different conditions were determined using the Wald test with a cut-off for absolute log2 fold change greater than 0.5 and adjusted *p*-values less than 0.05. Volcano plots and pathway analyses were generated with Ingenuity Pathway Analysis (IPA) (Qiagen), and the heatmaps were generated using RStudio's Shiny app.

### Statistical analyses

The statistical analyses in Figures 3, 7 and Supplementary Figure S1 were to compare levels for quantitative data. In the cases where the comparisons we wanted to test were based on the science (and not on the data) *t*-tests were used. In the case of Supplementary Figure S1, since the data are pre and post pump and hence paired, paired *t*-tests were used. We also controlled for multiplicity when appropriate. Tukey HSD was used in comparing all pairs of levels; this is the most conservative. When the aim was to compare each level to the control then Dunnett's test was used. We considered whether to use nonparametric tests in lieu of *t*-tests: Wilcoxon rank sum instead of the *t*-test and Wilcoxon signed-rank instead of the paired *t*-test. The data were not sufficiently skewed to warrant using the nonparametric analogues particularly since the sample sizes were modest. For all analyses, *p*-values were two-sided, and *p* < 0.05 was considered significant. Data are expressed as means ± standard error of mean (SEM), unless noted. Graphs were prepared in Graph Pad Prism.

## Results

### Aortic stenosis is associated with decreased SERT and increased HTR expression

We previously reported decreased SERT activity and over-representation of 5-HTTLPR LL genotypes in patients with degenerative mitral regurgitation (7). Thus, we first tested the expression of SERT and HTRs as well as the 5-HTTLPR genotypes in human explants from patients with AS and anatomically normal AV. Genotypes were labeled according to the length of the 5-HTTLPR region, “L” for long and “S” for short. The expected distribution of LS, LL and SS in the general population would be ≅50/25/25% according to the Hardy-Weinberg equilibrium.

Sixty-six patients undergoing aortic valve replacement (AVR) for severe AS were prospectively enrolled, along with 12 patients with anatomically normal AV (rejected cardiac donors with healthy valves) (Table 2). In patients with AS, median age was 67.5 [IQR 62.0–75.8], 30.3% NYHA Class III/IV, with a non-significant overrepresentation in LL and LS 5HTTLPR polymorphism relative to predicted Hardy-Weinberg equilibrium (LS 54.5%/ LL 28.8%/ SS 16.7%). In comparison 33% of patients (75 out of 225) undergoing MV replacement were carrying the LL 5HTTLPR polymorphism in our recent study, suggesting that AS progression has a weaker association than MR with SERT polymorphism genotype.

**Table 2:** Demographics and relevant medical history for all 66 aortic stenosis patients.

Parameter	Total (*n* = 66)
5-HTTLPR Polymorphism	
LL	19 (28.8%)
LS	36 (54.5%)
SS	11 (16.7%)
Men	38 (57.6%)
Women	28 (42.4%)
Age at surgery, *years* (median, *IQR*)	67.5 [62.0, 75.8]
Mitral Valve Dysfunction	3 (4.5%)
BAV	29 (43.9%)
Coronary Artery Disease	32 (48.5%)
Atrial Fibrillation	8 (12.1%)
NYHA Class III–IV	20 (30.3%)
Medications	
Aspirin	45 (68.2%)
SSRI	7 (10.6%)

**Figure 1:**
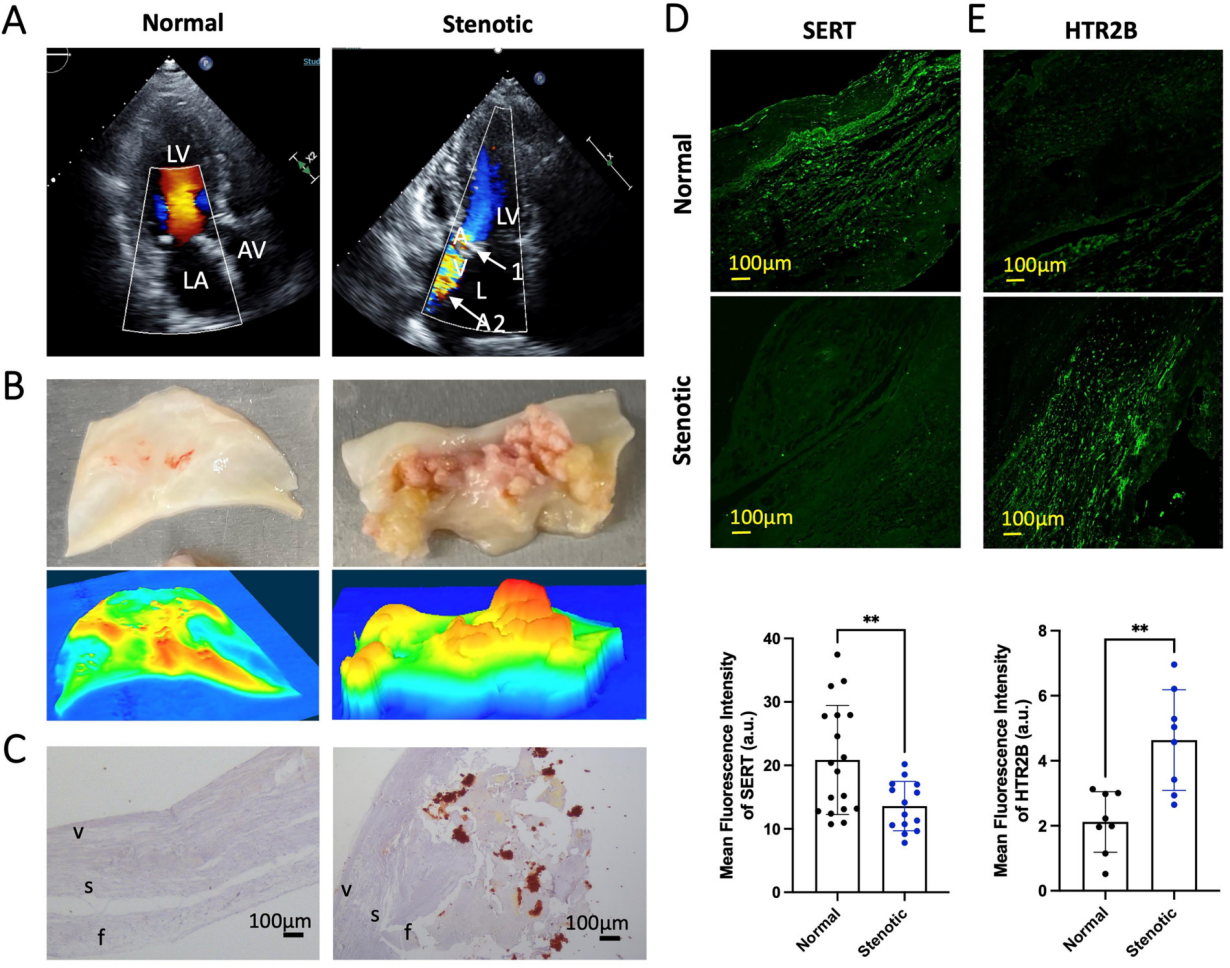
Phenotype of calcified AS aortic valve leaflets, includes *SERT* expression downregulation and *HTR2B* upregulation **(A)** representative echocardiography images of a patient with AS prior to surgical aortic valve replacement; LA, left atrium; AV, aortic valve; LV, left ventricle. Arrow 1 (top panel, white) indicates the thickened and calcified aortic valve. Arrow 2 (top panel, white) indicates AS jet visualized by Doppler ultrasound. **(B)** Resected aortic leaflet of human normal AV and stenotic AV (top). Representative topographic images generated of normal AV and stenotic AV by 3-Dimensional KEYENCE Laser profiler (bottom). Colors corresponding from high to low respectively: red-orange-yellow-green-blue. **(C)** Alizarin Red staining of normal AV samples from heart donors (left) and AS AV leaflets resected during surgery (right); f, zona fibrosa; s, zona spongiosa; v, zona ventricularis. Alizarin Red shows accumulation of calcium deposits (red/orange). The images were taken under brightfield microscope a 20× objective. **(D)** Representative immunofluorescence staining of SERT and the quantification of mean fluorescent intensity in normal AV and AS AV tissue (green). **(E)** Representative immunofluorescence staining of HTR2B receptor and the quantification of mean fluorescent intensity in normal and AS AV tissue (green). All immunofluorescent images were taken with a confocal microscope at 20× magnification. Error bars indicate SD. **Indicates *p-value* < 0.05 with unpaired Student's *t*-test.

We then assessed, at protein and RNA level, the expression of key 5HT signaling markers in patients with isolated AS (representative echocardiography images are shown in Figure 1A). In Figure 1B, we show resected human aortic leaflets from normal AV and stenotic AV (top panels) while representative topographic images generated by 3-Dimensional Laser profiler are shown in the bottom panel, highlighting the severity of AS in these patients. Alizarin red staining of explanted AV leaflets of AS patients and control showed pathological remodeling of the cusp with severe calcium accumulation (Figure 1C). Immunofluorescence staining of AV tissue indicated decreased SERT in AS vs. normal cusps (Figure 1D) and increased protein expression of HTR2B (Figure 1E). These results, mirroring our results on degenerative mitral regurgitation, led us to hypothesize that 5HT might be able to exert increased HTR-dependent signaling in AS due to decreased clearance of extracellular serotonin by reduced SERT.

**Figure 2:**
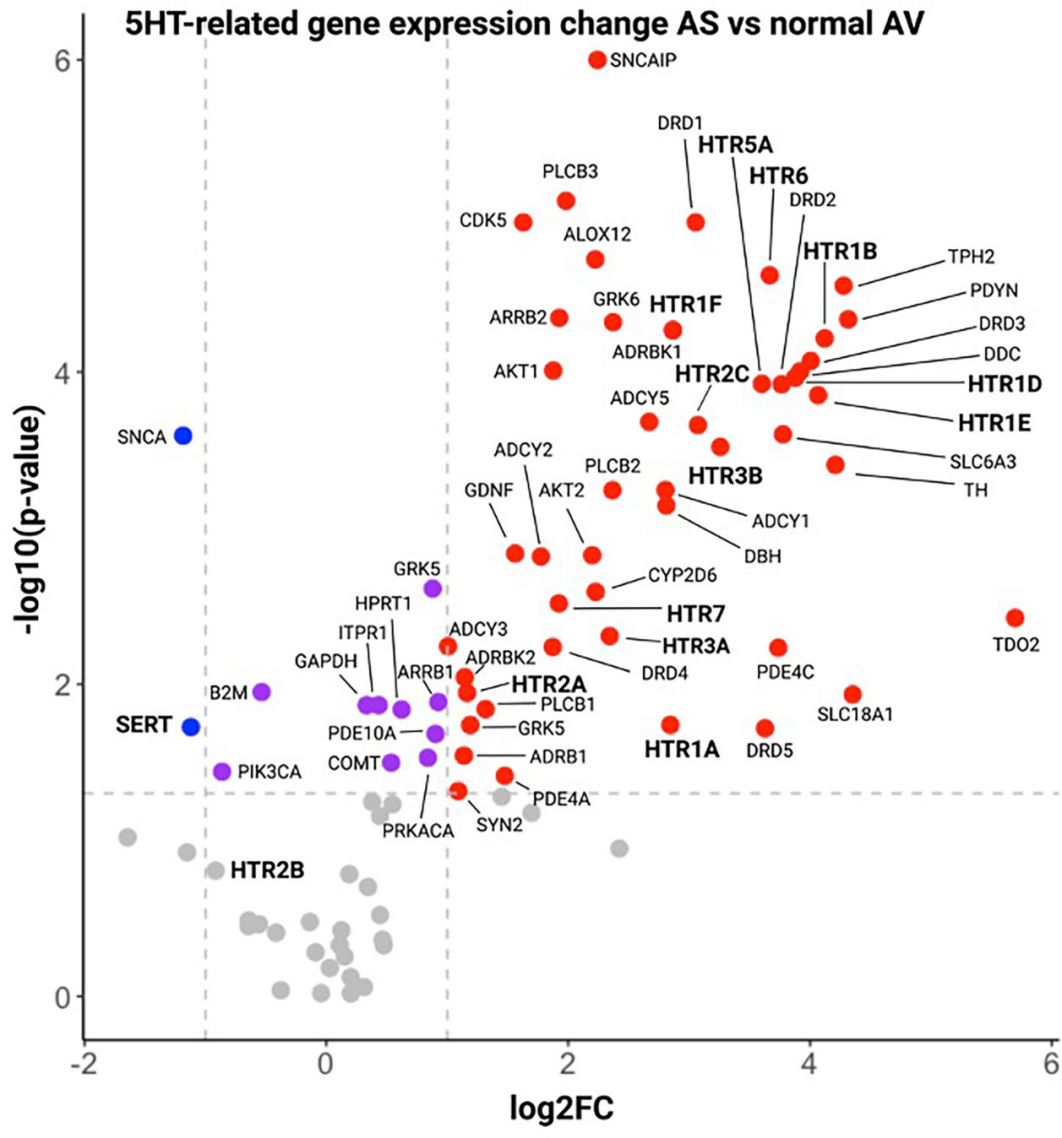
Volcano plot of gene expression results by RT2 profiler panels of 5HT-related genes in aS patient vs. control AV tissue. Serotonin-related genes in Stenotic AV tissue in comparison with normal AV tissue. Horizontal dotted lines denote the genes with −log10 > 1.3 (corresponding to a *p* value = 0.05 by Student t test, purple dots), vertical dotted lines define genes with a log2 fold change >1 (red dots) or <−1 (blue dots), corresponding to fold change = 2 and 0.5 vs. normal, respectively.

Using RT2 profiler gene arrays, we then compared gene expression in AS vs. normal AV patients. In AS, SERT was significantly downregulated compared to normal AV. Furthermore, 12 HTRs were significantly upregulated, including HTR2A and HTR2C (Figure 2). However, HTR2B expression was not significantly different in AS compared to normal AV at transcriptional level. Consistently, our analysis of an independent RNAseq dataset from AS patients (GSE153555) showed a similar pattern of SERT and HTR2B gene expression. Interestingly, other upregulated genes included AKT1 and AKT2, which are important for AngII-mediated cardiac hypertrophy. We, and others, have demonstrated a crosstalk between AngII and 5HT signaling with human and murine data showing that specific inhibitors of HTR2B mitigate AngII-induced MV remodeling (13). Thus, these results formed our rationale to mechanistically investigate modulation of 5HT-signaling in a murine model of aortic valve remodeling, namely via AngII infusion. We hypothesize that AS may be characterized by decreased SERT and increased HTR expression or activity, potentiating the effects of AngII, and creating a therapeutic target in HTR blockade.

### AngII-administered mice have aortic valve leaflet thickening, impaired hemodynamics, and fibrotic/osteogenic gene remodeling, each mitigated by HTR2B blockade

**Figure 3:**
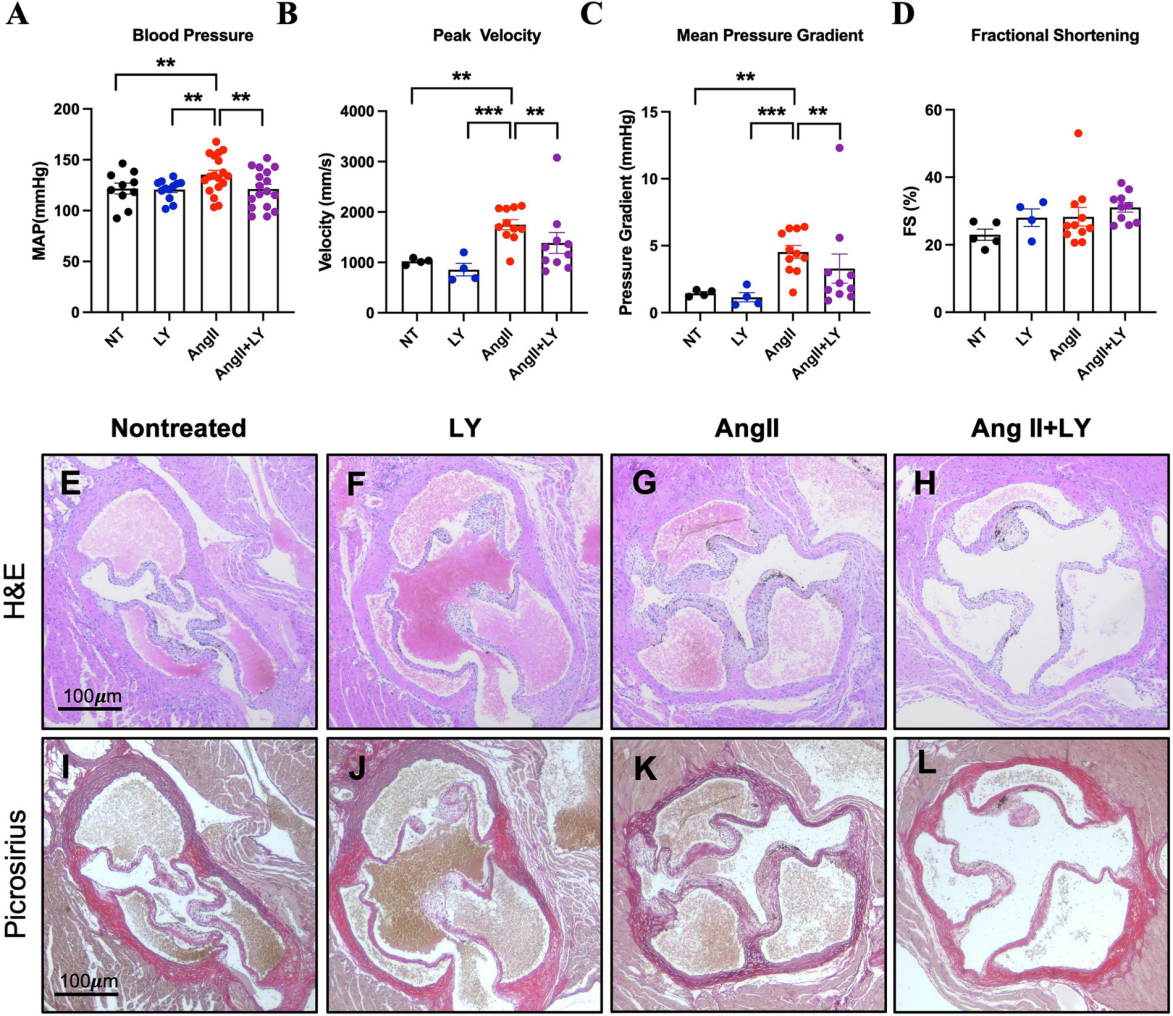
LY272015 prevents hypertensive response and aortic valve thickening. **(A)** Blood pressure measurements in NT (nontreated), LY, AngII and AngII+LY mice. **(B)** Peak AV velocity, **(C)** Mean AV pressure gradient, and **(D)** fractional shortening (FS) in NT, LY, AngII, and AngII+LY mice. Individual dots correspond to an average of ∼10 repeated blood pressure measurements in each mouse or individual mice for echo measurements. Representative H&E (**(E)**: saline-treated mice, **(F)**: WT mice treated with AngII, (1,000 ng/kg/min) using osmotic pumps (Alzet 2004) for 28 days; **(G)**: WT mice treated with LY272015 (3 mg/kg in sterile PBS) twice/week over the 28-days, **(H)**: WT mice treated with AngII, (1,000 ng/kg/min) using osmotic pumps (Alzet 2004) for 28 days also treated with LY272015 (3 mg/kg in sterile PBS) twice/week over the 28-days. Picrosirius red staining of nontreated **(I)**, AngII **(J)**, LY **(K)**, and AngII+LY **(L)** murine aortic valves. Arrow indicates 1/3 mice aortic valve semilunar leaflet. All stains, *n* ≥ 5/group. Error bars indicate SEM. **Indicates *p-value* <0.05 and ***indicates *p*-value <0.01 by ANOVA, Wilcoxon Rank Sum, or Student's t-test as appropriate.

We studied the role of HTR2B blockade in controlling AV remodeling *in vivo*. Chronic infusion of AngII in hypercholesteremic mice resulted in elevated blood pressure compared to control mice (AngII mean arterial pressure (MAP) 135.3 ± 18.9 mmHg vs. NT 121.7 ± 16.8, *p* < 0.05), and LY272015 prevented this MAP increase due to AngII (AngII+LY MAP 121.3 ± 18.8, *p* < 0.05 vs. AngII) (Figure 3A). On echocardiography, markers of aortic valve disease, increased velocities, and pressure gradients, were observed in AngII mice (AngII mean gradient 4.40 (IQR 3.6–6.1) mmHg vs. NT 1.5 (1.3–1.6), *p* < 0.05; AngII peak velocity 1,700.8 (IQR 1,604.7–2,068.8) mm/s vs. NT 1,022.0 (993.8–1,048.8), *p* < 0.05), without significant changes to fractional shortening (28.3% vs. 23.0%) (Figures 3B–D). LY272015 reduced both the peak velocity and mean pressure gradient (AngII+LY mean gradient 2.0 (IQR 1.5–3.0) mmHg and peak velocity 1,225.6 (1,014.6–1,415.1) mm/s, *p* < 0.05 vs. AngII), and these values were not significantly different from the NT gradients/velocities. All other echocardiographic parameters are shown in Supplementary Table S1.

Furthermore, AngII infusion in hypercholestrolemic mice resulted in remodeling of the cardiac structures, including thickening of the heart valves (Figures 3E–L). The choice of this animal model was driven by growing evidence indicating that AngII induces its pleiotropic effects through NADPH-driven generation of ROS. Twenty-eight days of AngII infusion led to pathological thickening of the aortic valve (Figures 3G,K). However, mice receiving concurrent 3 mg/kg LY272015 treatment, both alone (Figures 3F,J) and with AngII (Figures 3H,L), retained thin cusps more closely resembling not-treated mice.

To assess the confounding vasopressor effect of AngII on our hemodynamic results, a subset [*N* = 7–8 per group] of AngII+LY and AngII mice had their Alzet pumps removed, with blood pressure and echocardiography repeated 24–48 h later. After pump explant, blood pressure in both AngII and AngII+LY groups decreased significantly (AngII decrease 33.7 ± 12.9 mmHg, *p* < 0.05; AngII+LY 27.7 ± 16.0) but remained lower in AngII+LY vs. AngII (*p* < 0.05) (Supplementary Figure S2A). This was associated with a non-significant trend toward decreased gradients/velocities in both groups, although here too AngII+LY had 14%–17% reduction in mean gradient/peak velocity), similar to the results observed prior to pump removal (Supplementary Figure S2).

**Figure 4:**
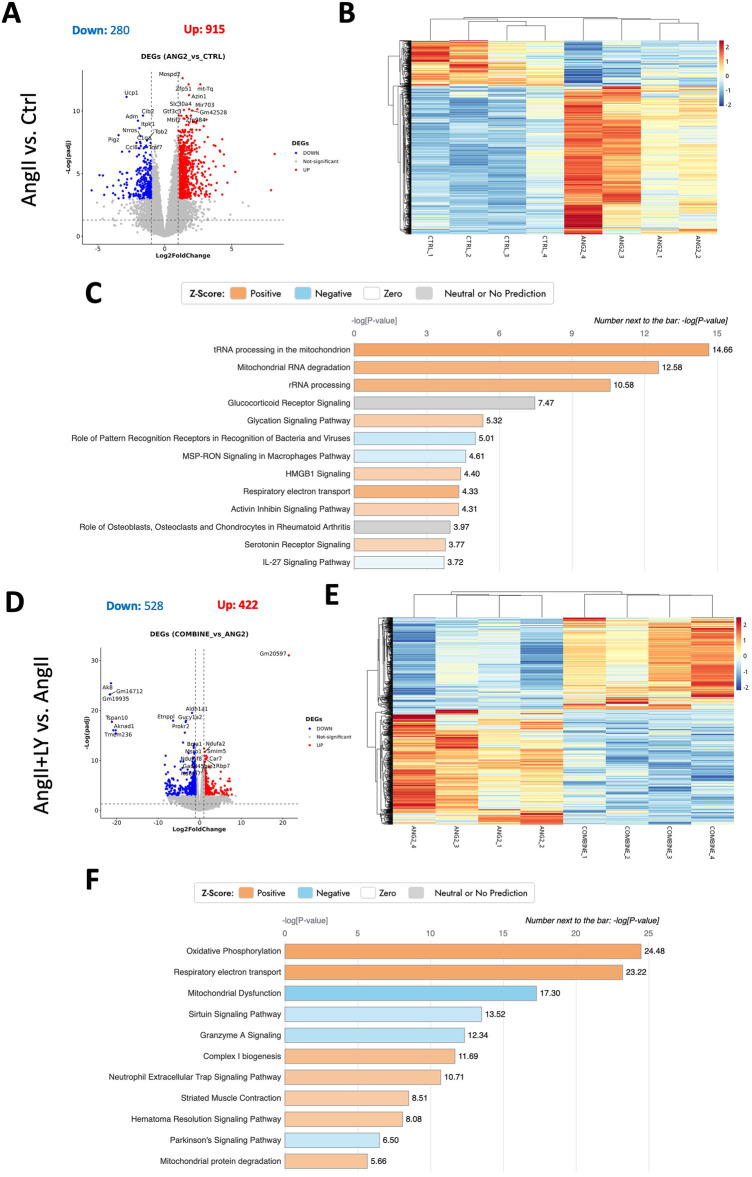
Bulk RNA sequencing reveals the reversal of AngII-induced remodeling in mouse AVs via the treatment with HTR2B antagonist, LY272015. **(A)** Volcano plot of Differentially Expressed Genes (DEGs) number, *p-value* <0.05 & |log2foldchange| > 1, of the AngII-treated group compared to the Ctrl group. **(B)** Heatmap showing distribution of DEGs among different samples. **(C)** Ingenuity Pathway Analysis showing top differentially regulated pathways (-log(*p*-value) > 0). **(D)** Volcano plot of Differentially Expressed Genes (DEGs) number, *p-value* < 0.05 & |log2foldchange| > 1, in the AngII+LY-treated group compared to the AngII group. **(E)** Heatmap showing distribution of DEGs among different samples of AngII+LY and AngII groups. **(F)** Ingenuity Pathway Analysis showing top differentially regulated pathways (-log(*p*-value) > 0) in the AngII+LY-treated group compared to the AngII group. Z-scores indicate the likelihood of pathway activation or inhibition. Positive Z-score indicates activated and Negative Z-score indicates inhibited pathways.

**Figure 5:**
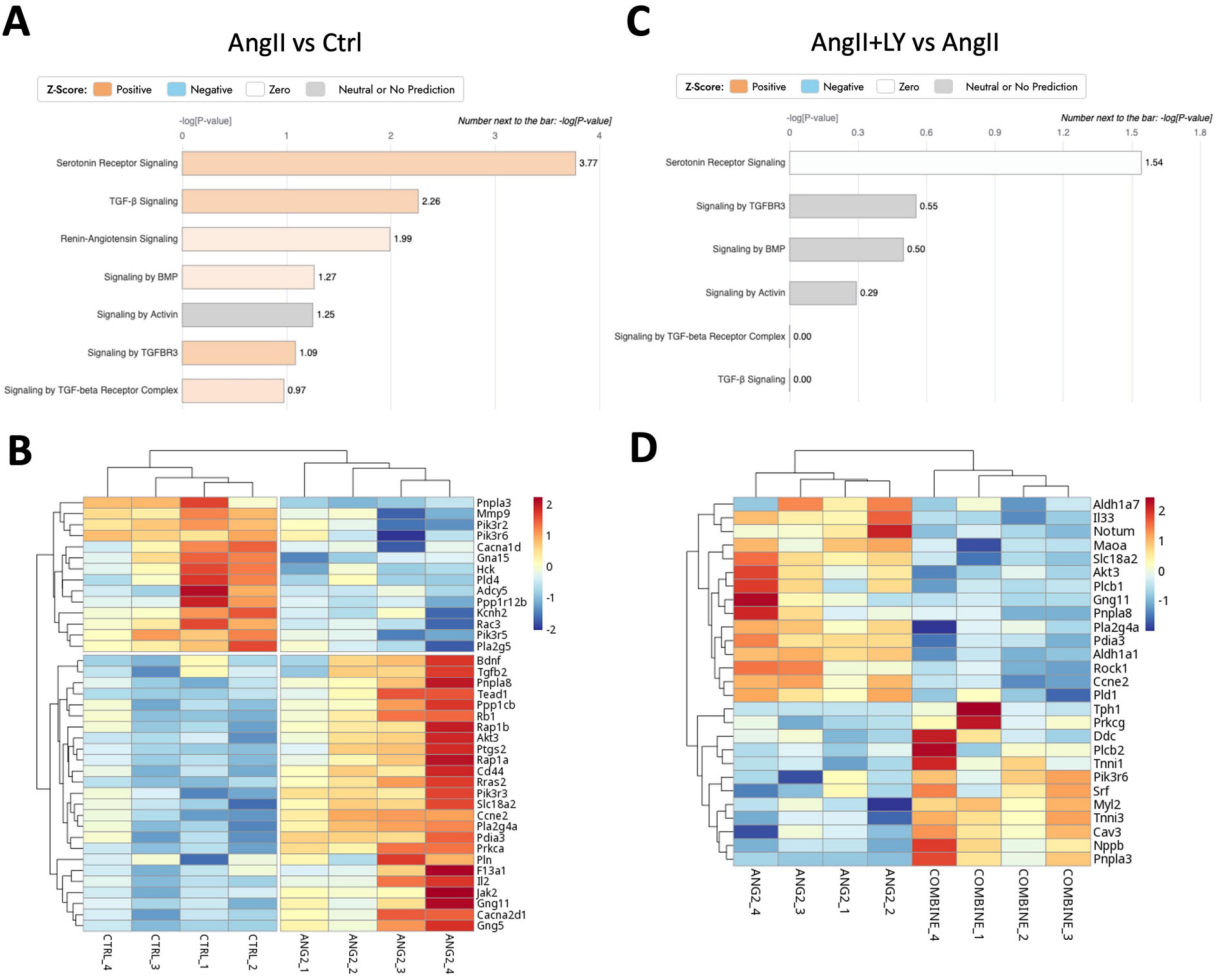
LY272015 reverses AngII-induced gene expression of serotonin signaling in mouse AVs. **(A)** Ingenuity pathway analysis showing upregulation of AngII-induced differentially regulated Serotonin and TGFβ signaling pathways compared to the Ctrl group. **(B)** Heatmap showing differential expression of Serotonin-related genes between AngII-treated and Ctrl groups. **(C)** Ingenuity pathway analysis showing downregulation of AngII+LY induced differentially regulated Serotonin signaling pathway compared to the AngII induced group. **(D)** Heatmap showing differential expression of Serotonin-related genes between the AngII+LY and AngII alone group. Z-scores indicate the likelihood of pathway activation or inhibition. Positive Z-score indicates activated and Negative Z-score indicates inhibited pathways.

**Figure 6:**
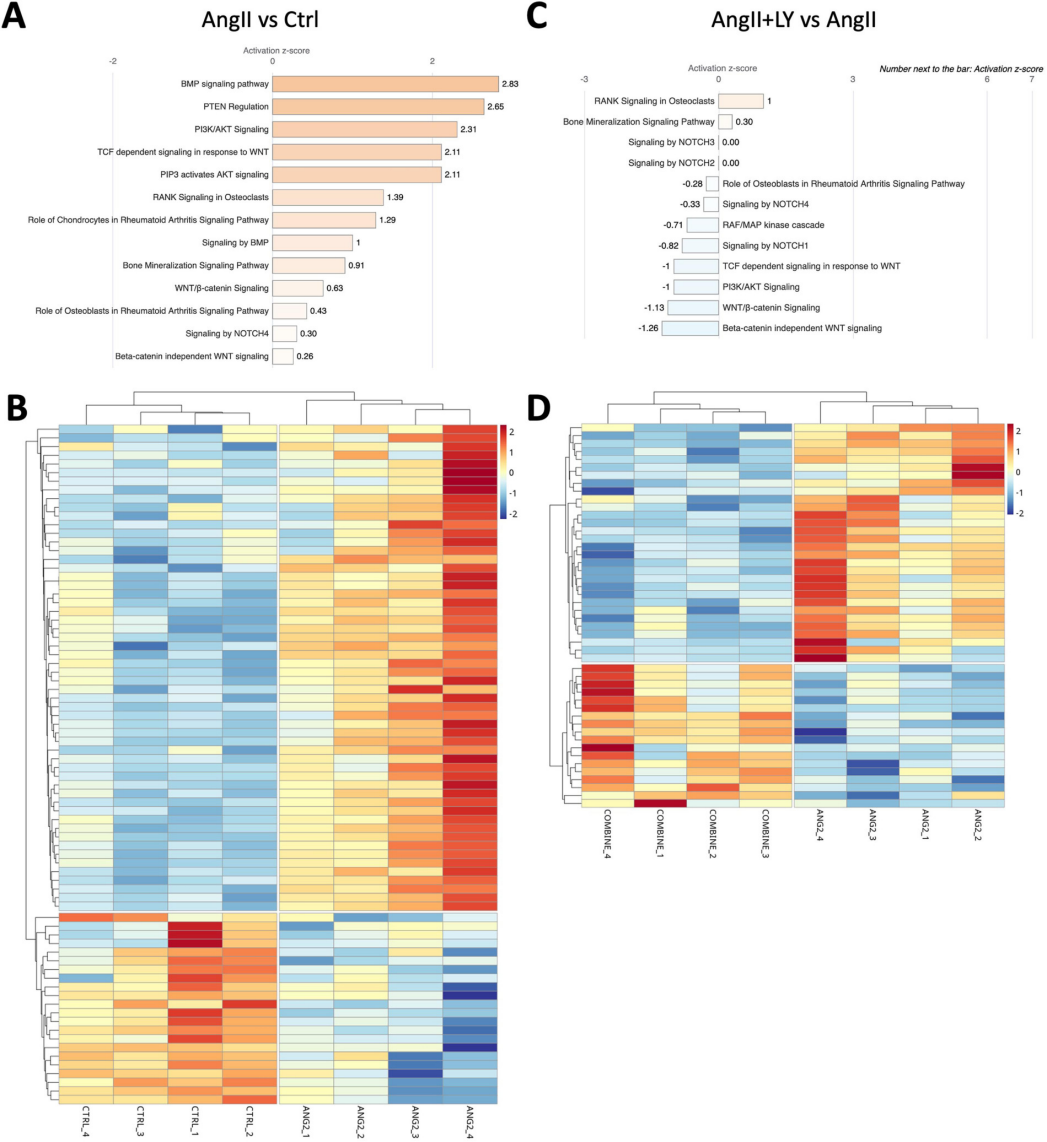
LY272015 reverses AngII-induced gene expression of calcification-related signaling in mouse AVs. **(A)** Ingenuity pathway analysis showing upregulation of AngII-induced differentially regulated Calcification-associated signaling pathways compared to Ctrl group. **(B)** Heatmap showing differential expression of Calcification pathway genes between AngII-treated and Ctrl groups. **(C)** Ingenuity pathway analysis showing downregulation of AngII+LY induced differentially regulated Calcification-associated signaling pathways compared to the AngII group. **(D)** Heatmap showing differential expression of Calcification pathway genes between the AngII+LY and AngII alone group. Z-scores indicate the likelihood of pathway activation or inhibition. Positive Z-score indicates activated and Negative Z-score indicates inhibited pathways.

Bulk RNA sequencing of the explanted AVs showed the greatest differences between AngII and untreated Ctrl mice (1,195 differentially expressed genes, DEGs) (Figures 4A,B; Supplementary Figure S3). AngII activated important signaling axes that are known to play key roles in the development of AS such as mitochondrial tRNA processing and RNA degradation, rRNA processing, glycation signaling, HMGB1 signaling, and 5HT signaling pathways (17) (Figure 4C). On the other hand, combined treatment (AngII+LY) compared to AngII treatment alone differentially regulated 950 genes (422 up and 528 down) (Figures 4D,E). The treatment of LY272015 alone did not induce any effect as expected; however, its combined treatment with AngII partly reversed the detrimental effects of AngII (Figure 4F**)**. LY272015 treatment positively regulated mitochondrial signaling pathways that are associated with physiologic heart valve function such as improved oxidative phosphorylation (18), improved respiratory electron transport and reduced mitochondrial dysfunction (19), and reduced granzyme signaling (20, 21). In addition, we found that TGFβ signaling-associated pathways, including fibrosis and extracellular matrix (ECM) associated genes, were significantly enriched with AngII treatment (Figures 5A,B; Supplementary Figures S4A,B), suggesting an enhanced fibrosis and stiffening of aortic valves. On the other hand, AngII+LY treatment suppressed these gene pathways including the 5HT signaling-related genes (Figures 5C,D; Supplementary Figures S4C,D). More interestingly, aortic calcification and chondrogenesis-related signaling, such as PTEN (22), PI3K/AKT (23–25), Wnt/β-catenin (26, 27), BMP (17, 28), and Notch (29, 30) pathways, were moderately upregulated with AngII treatment (Figures 6A,B), which was effectively reversed by the addition of LY (Figures 6C,D). Besides, our disease association analyses on DEGs revealed that the gene network activated by AngII treatment was associated with activation of inflammatory signaling and bone cell differentiation (Supplementary Figure S5) while LY treatment was largely associated with positive regulation of muscle function and negatively associated with fibrosis and cardiovascular diseases (Supplementary Figure S6), suggesting a relationship to a potential therapeutic role of HTR2B inhibition via LY272015.

In summary, these results in mice show that pathological remodeling occurs in response to AngII, with valve thickening, increased velocities/gradients, and cellular genetic changes mimicking changes seen in AS. HTR2B blockade by LY272015 prevents histologic thickening, with improved hemodynamics, mitigation of VIC activation, early calcific remodeling, and TGFβ-mediated pro-fibrotic signaling.

### Under decreased SERT conditions in cell culture, AngII leads to increased HTR2B expression, VIC activation, and fibrotic remodeling in human AVIC

**Figure 7:**
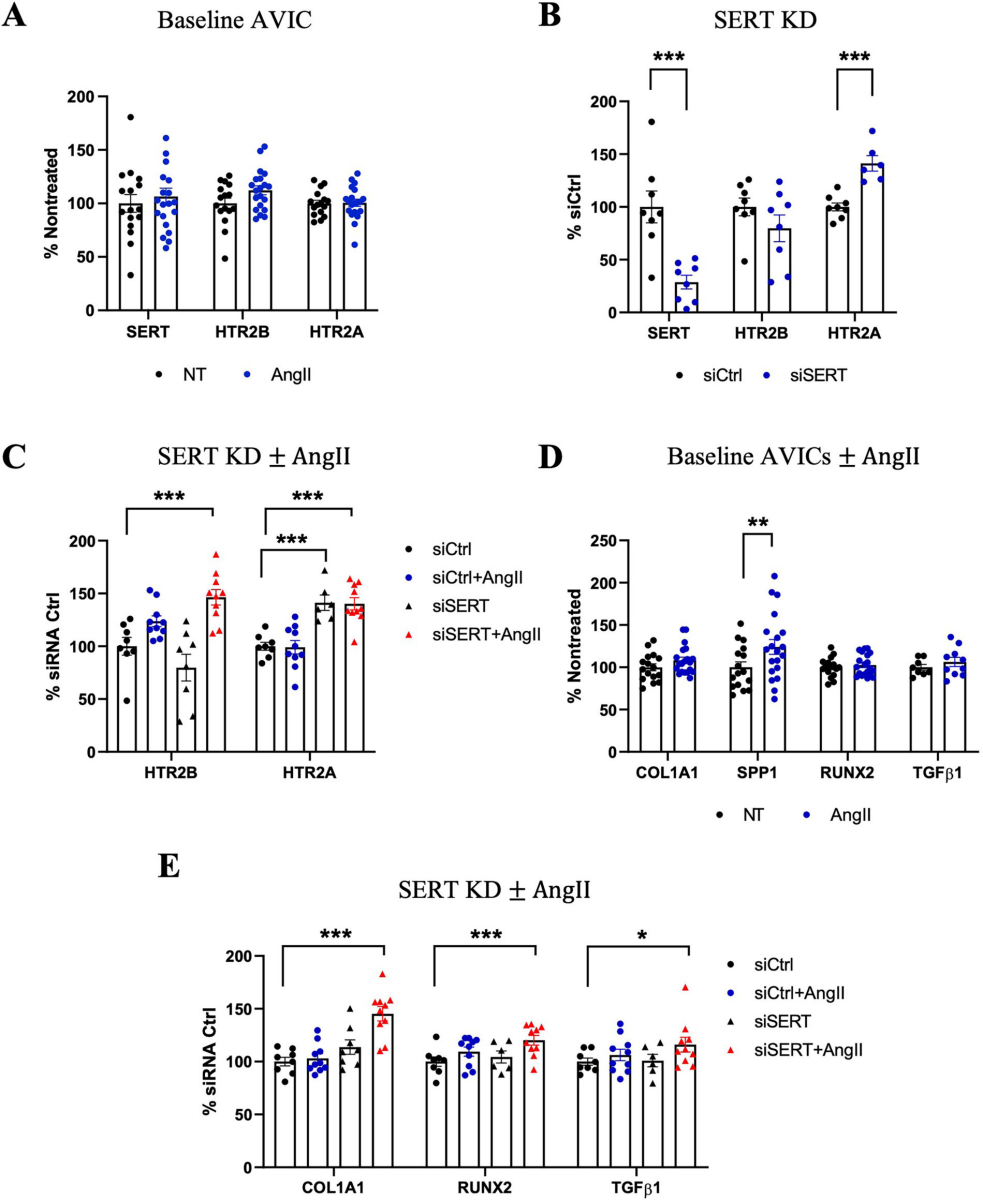
Effects of AngII on human AVICs with SERT knockdown. **(A)** Gene expression of SERT, HTR2B, HTR2A in AngII-treated compared to Nontreated (NT) Ctrl AVICs. **(B)** SERT KD by siRNA lead to increased expression of HTR2A gene expression. **(C)** HTR2A and HTR2B expression with siSERT or siSERT combined with AngII treatment. **(D)** COL1A1, SPP1, RUNX2, TGFβ1 expression in response to AngII treatment in human AVICs compared to Nontreated group. **(E)** COL1A1, RUNX2, TGFβ1 expression treated with siSERT alone or siSERT combined with AngII treatment. All gene expression results were calculated by the 2^−ΔΔCT^ method, *n* ≥ 4 per group. Error bars indicate SEM. *P-value* vs. NT. *indicates *p-value* <0.1, **indicates *p-value* <0.05, and ***indicates *p*-value <0.01 by Student's *t*-test or one-way ANOVA with *post-hoc* Dunnett's test.

Oxidative stress and mitochondrial dysfunction are closely related and are associated with the progression of AS (18, 31–33). Having established that AngII treatment in mice largely impacted mitochondrial function and oxidative phosphorylation (Figure 4), we tested the effects of oxidative stress by H_2_O_2_ and AngII/5HT signaling on the aortic valve using human aortic valve interstitial cells (AVICs) cultured from normal human AV. Oxidative stress with H_2_O_2_ alone decreased SERT and increased HTR2B expression (Supplementary Figure S7). In contrast, AngII treatment did not significantly alter SERT or HTR2B expression, and neither H_2_O_2_ or AngII changed HTR2A expression (Figure 7A; Supplementary Figure S7). siRNA-mediated SERT knockdown resulted in a sustained decrease in SERT expression. SERT knockdown alone increased HTR2A expression but did not significantly alter HTR2B. However, the combination of SERT knockdown plus AngII significantly increased HTR2B expression. HTR2A expression remained elevated after SERT knockdown and did not change significantly with subsequent AngII stimulation (Figures 7B,C). Thus, oxidative damage may potentiate 5HT signaling via lowered SERT and increased HTR2B. Moreover, lowered SERT may exacerbate the response to AngII, either through increased HTR2B or other means.

The downstream effects of VIC activation and osteogenic marker expression due to AngII treatment were examined, both alone and after SERT silencing. AngII treatment led to increased SPP1 levels, correlating well with our murine experiment, but did not change expression of COL1A1, RUNX2, and TGFβ1 (Figure 7D). However, in the setting of SERT knockdown, which independently causes either none or mild upregulation of these genes, siSERT + AngII led to upregulation of COL1A1 and RUNX2, and a trend toward increased TGFβ1 (Figure 7E). These results indicate the sensitivity of AVICs to AngII signaling induced remodeling, particularly under altered SERT conditions.

## Discussion

In this study, we demonstrate that AS involves altered 5HT signaling and that the pathological AngII-induced aortic valve response in mice, modeling early non-calcified fibrotic remodeling, is serotonergically mediated. This was accomplished through AngII treatments in two models, our murine *in vivo* model involving HTR blockade and our human AVIC *in vitro* model involving SERT siRNA. AngII infusion led to fibrotic thickening in murine AVs, a narrowing of the AV orifice measured by increased velocities and gradients, and gene expression changes involving 5HT, TGFβ, Wnt/β-catenin, PI3K/AKT, and Notch signaling pathways, which are involved in fibrosis and early stages of calcification of aortic valves. Previously, HTR2B signaling has been shown to be the key driver of valvular heart disease and its inhibition mitigated valvular pathology (3, 7, 34). Consistently, HTR2B blockade in our study using the selective HTR2B antagonist, LY272015, which retained thin cusps, decreased velocities and pressure gradients, and reversed the RNA expression profile induced by AngII treatment in mice. Although impact of LY272015 *in vivo* is mainly mediated through its antagonism for HTR2B, which is dominantly expressed in heart valves, given the lack of *in vivo* pharmacokinetic data at the selected drug dose, it may be possible that protective effect of LY272015 is partially driven by its antagonism on other HTR2 receptors, e.g., HTR2A, expressed in AV tissue.

Acute oxidative stress by H_2_O_2_ in human AVICs led to SERT downregulation and HTR2B upregulation, and AngII stimulation in the setting of SERT knockdown was associated with VIC activation and fibrotic remodeling, and early pro-calcific remodeling in association with increased expression of SPP1, RUNX2, and COL1A1. Although AngII is known to induce oxidative stress, AngII alone did not affect SERT expression. This is likely due to distinct mechanisms of AngII and ROS. AngII works through AT1 receptors and regulates GPCR-dependent pathways (e.g., MAPK, PKC) and do not directly involve in transcriptional or post-transcriptional mechanisms that directly regulate SERT expression. Indeed, a previous study (35), although in different cell type, has shown that AngII does not regulate SERT expression but reduces its 5HT reuptake through direct competition with 5HT. In contrast, H_2_O_2_ directly induces oxidative stress, which robustly activates redox-sensitive transcription factors that may potentially regulate SERT expression (36–38). Indeed, 5HT signaling is known to be sensitive to the oxidative stress making SERT potentially susceptible to downregulation under oxidative stress with H_2_O_2_ (39, 40). These suggest that redox imbalance is more dominant regulator of SERT expression than AngII alone while AngII potentially regulates SERT function and not its expression. The clinical relevance of these scenarios, increased HTR signaling and decreased SERT expression and function, was examined through our large cohort of patients with severe AS, whose valves displayed significant upregulation of many HTRs and downregulation of SERT.

Prior studies, by us and others, have examined the cellular pathways involved in 5HT-AngII mediated heart valve disease. Both HTR2A and HTR2B signaling upregulate TGFβ signaling thereby increasing extracellular matrix (ECM) protein expression and activating valve interstitial cells (VICs) (3, 4). Mice lacking SERT have increased fibrosis of the mitral and aortic valve leaflets, as well as cardiac fibrosis and decreased cardiac function (41). SERT inhibition by SSRIs lead to increased HTR2B expression (7). In MR, HTR2B is upregulated whereas SERT is downregulated (7, 42, 43). Moreover, we observed that HTR2B protein levels are more prominently impacted than mRNA levels in AS. Although its mechanism is still unknown, increased HTR2B protein in the absence of changes in mRNA suggests a post-transcriptional regulation, such as enhanced translation, reduced receptor degradation, or altered trafficking and merits further research to mechanistically dissect HTR2B-mediated valvular remodeling.

AngII, through the AT1 receptor (AT1R), leads to cardiac hypertrophy and valve thickening through generation of ROS through NADPH oxidase and increased TGFβ-1 signaling (4, 44, 45). Moreover, crosstalk exists, at least within cardiomyocytes, such that cytokine release due to either AngII/AT1R or 5HT/HTR2B activation is prevented by blockade at the other receptor (9). While previous research has explored AS, the specific impact of reduced SERT expression and HTR2B activation within the context of AngII-driven pathways in the aortic valve has not been thoroughly investigated. In this study, we define the role of SERT in aortic valve pathology at early stages of AS, showing that it is downregulated in valve tissue and modulated partly by AngII and oxidative stress in human AVICs. We also demonstrated that HTRs are upregulated in diseased valves and that blocking HTR2B has therapeutic potential to attenuate progression of AS in early profibrotic phases. AngII induces fibrotic remodeling in AV, which is intensified by SERT suppression and alleviated by HTR2B blockade.

Moreover, our results provide further understanding about established markers of aortic valve disease and AVIC activation, and demonstrate how these are 5HT-sensitive. RUNX2, a transcription factor and marker of osteogenic-like transdifferentiation in AVICs, is upregulated in response to oxidative stress, and is involved in AV calcification with upregulation in AS [10, 12]. Herein, RUNX2 increased in AVICs in response to siSERT + AngII, and the increase observed in AngII mice was prevented by HTR2B blockade via LY272015. SPP1, or Osteopontin, is upregulated in AS, serves as a marker of VIC activation, and may provide a compensatory mechanism to mitigate AV calcification (11, 14, 46). We also demonstrated that SPP1 increased due to AngII in both human-derived AVICs and murine AVs, and this was mitigated by HTR2B inhibition. Finally, AngII activates phospholipase A2 and the arachidonic acid pathway, that is implicated in inflammation, AV calcification and ROS release (45, 47). Our results show this occurs in AngII infused mice developing AV disease and that LY272015 mitigates this activation. Finally, beyond the cellular changes, this study provides histological and hemodynamic data to better evaluate the clinical consequences of these changes.

The authors acknowledge that our study had several limitations. First, the *in vitro* AVIC cell culture studies did not compare AVICs isolated from AS cases to normal hearts obtained at the time of transplantation. It was rather limited to comparison of patient valve tissue which is mostly populated by AVICs. Additionally, the direct contributions of 5HT on calcification–a major hallmark of AS disease progression–were not assessed, limiting mechanistic insight into its contribution to valvular mineralization, and remains an important area for future research. The AngII-induced model on wildtype mice also lacks established relevance to late onset of calcific aortic valve disease in mice. However, gene expression changes demonstrated moderately activated fibrotic and calcification signaling by AngII, suggesting that AngII involved in relatively early stages of aortic remodeling, and our findings may rather represent the role of 5HT signaling in mild aortic stenosis. Future studies involving more relevant animal models for late-stage calcified AS would be necessary to explore the complete role of 5HT signaling in AS pathology. Furthermore, our analyzes on 5HT signaling were conducted on valve specimens from a relatively small number of patients, which may affect the generalizability and statistical power of our findings. Indeed, the limitation in patient number led to insignificant association of 5HTTLPR SERT polymorphism genotypes with AS. Thus, future studies on larger cohort of patients are required to assess potential role of SERT polymorphisms in AS progression and development. Despite these limitations, the endpoints and results of the present experiments provide meaningful evidence supporting a major role of 5HT signaling pathways in aortic valve pathology.

Overall, our results lead to several clinical implications. Currently, despite our knowledge of AngII-induced valvular changes, no therapy exists to prevent or slow the progression of AS. AT1R blockade, indicated in hypertension, heart failure, and coronary artery disease, has not been shown to prevent AS progression (48–51). Increased ROS activity and reduced SERT expression are present in the diseased AV, and our *in vitro* studies show that AngII, potentially through increasing ROS, impacts SERT/HTR2B axis and valvular remodeling, yet further mechanistic studies are needed to establish connection between AngII and ROS in regulating SERT and HTR2B function in AS. Moreover, our *in vivo* study shows that the pathological effects of AngII can be mitigated by preventing HTR2B signaling. Thus, HTR2B blockade with LY272015 is a promising therapeutic target to attenuate disease mechanisms, partly mediated by AngII, in the progression of aortic valve disease.

## Conclusions

This study established a mechanistic link between 5HT signaling and AngII-mediated AV remodeling, highlighting a central role for reduced SERT expression and heightened HTR2B activation in the pathogenesis of AS. Through complementary *in vivo* and *in vitro* models, we demonstrated that AngII promotes fibrotic and early calcific responses in AVs, which are amplified by SERT downregulation and effectively attenuated by pharmacological HTR2B inhibition. These findings were corroborated by transcriptomic changes, hemodynamic impairments, and increased expression of key markers such as RUNX2 and SPP1, all reversed with LY272015 treatment. Furthermore, analysis of human AV tissue revealed consistent downregulation of 5HT pathway components in AS, underscoring the translational relevance of our results. Taken together, our data identify HTR2B as a potential therapeutic target in the prevention or treatment of valvular disease, partly mediated by AngII, and a compelling rationale for further clinical investigation of serotonergic modulation in AS.

## Data Availability

The original contributions presented in the study are publicly available. This data can be found in the GEO database (https://www.ncbi.nlm.nih.gov/geo/), accession number: GSE318212.
