# Supplementary material for: Serotonin transporter downregulation is associated with aortic stenosis, and early profibrotic remodeling is mitigated by pharmacological inhibition of HTR2B receptor
Source: Front Cardiovasc Med. 2026 Feb 12;13:1729078. doi: 10.3389/fcvm.2026.1729078 (PMC12935953; doi:10.3389/fcvm.2026.1729078)
Supplement: Supplementary file 1 [file Datasheet1.pdf]

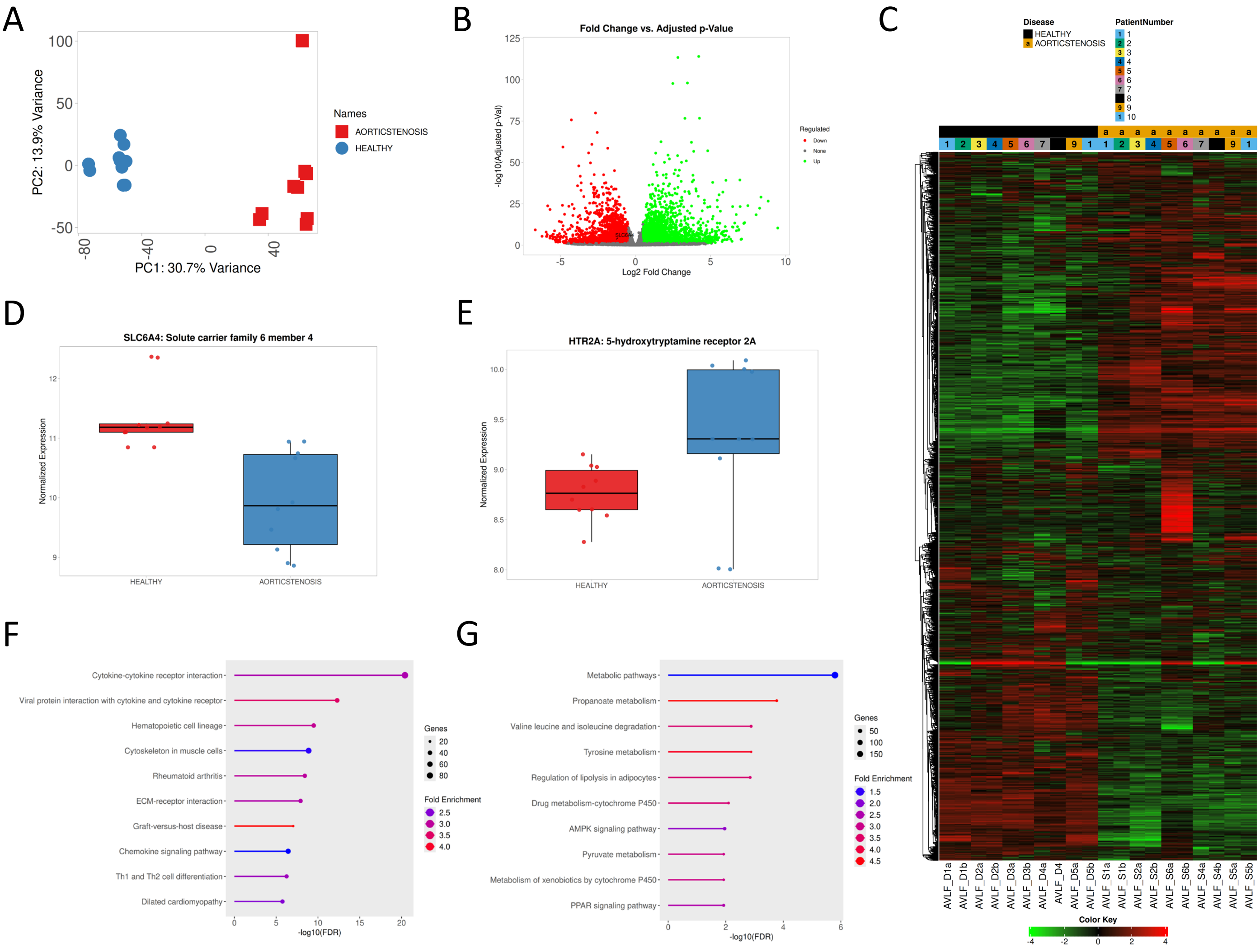

Figure S1. Analysis of aortic stenosis bulk RNAseq dataset (GSE153555). A) PCA analysis of Healthy vs AS valves (n=10 per group). **B)** Differentially up (2503) and downregulated (2244) genes (DEGs) in AS compared to Normal aortic valves (**p-adj** < 0.05 by Wald test; log2FC > 0.5). **C)** Heatmap showing expression of top 2000 DEGs. **D)** Downregulation of SERT (SLC6A4) gene expression in AS patients. **E)** Upregulation of HTR2A in AS. HTR2B expression did not pass the minimum threshold. **F-G)** Significantly enriched pathways (FDR < 0.05) of upregulated (F) and downregulated (G) DEGs.

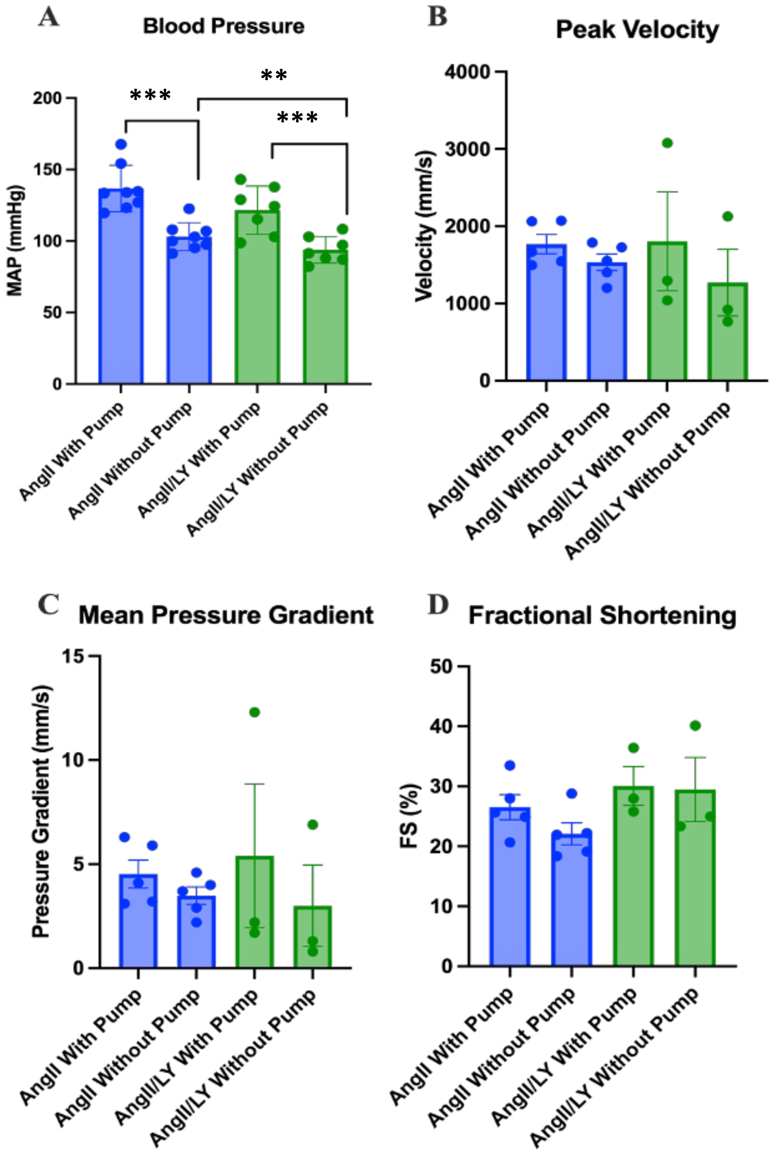

Figure S2. Blood pressure and echocardiography before and after pump removal. (A) Blood pressure; (B) peak AV velocity, (C) mean AV pressure gradient, and (D) fractional shortening in AngII and AngII/LY mice prior to pump removal and post pump removal. Individual dots correspond to individual mice. Data is shown as mean±SEM. **indicates *p-value <0.05* and ***indicates *p-value <0.01* by paired t-tests.

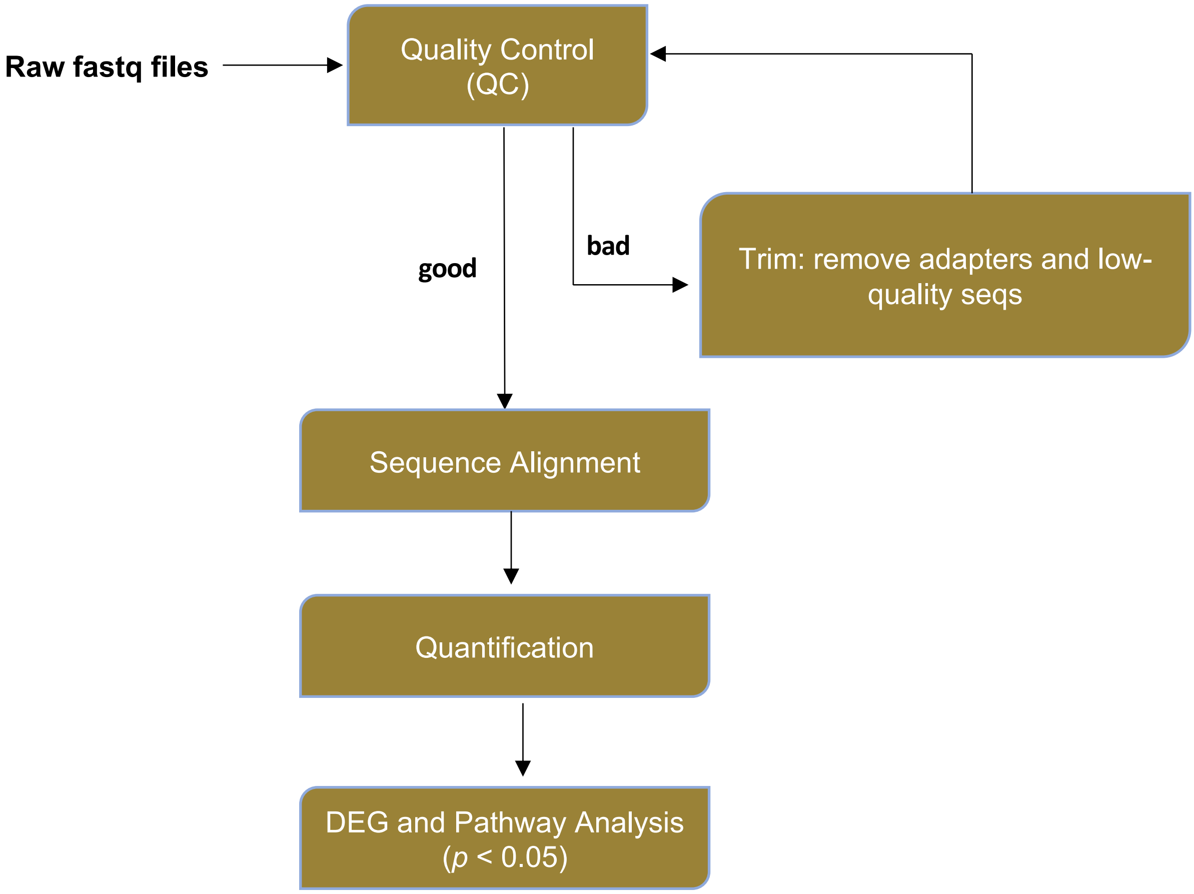

Figure S3. RNAseq analysis workflow

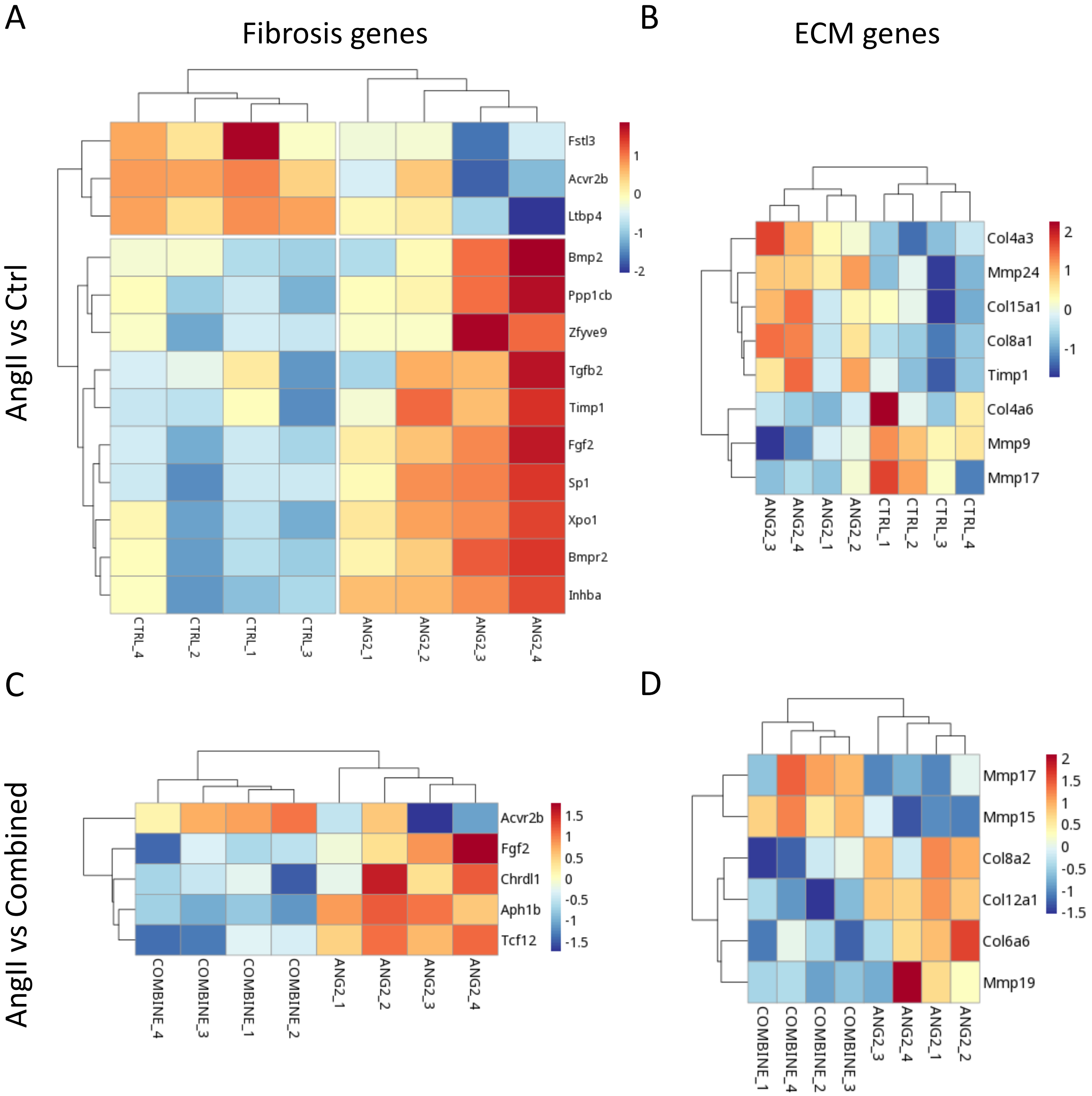

Figure S4. Heatmaps of statistically significant fibrosis and ECM-related genes in mouse AVs. (**A-B**) Heat maps showing fibrosis (A) and ECM (B) gene expression among the DEGs in AngII-treated mice compared to Ctrl group. (**C-D**) Heat maps showing fibrosis (AC and ECM (D) gene expression among the DEGs in AngII+LY-treated mice compared to AngII-treated group.

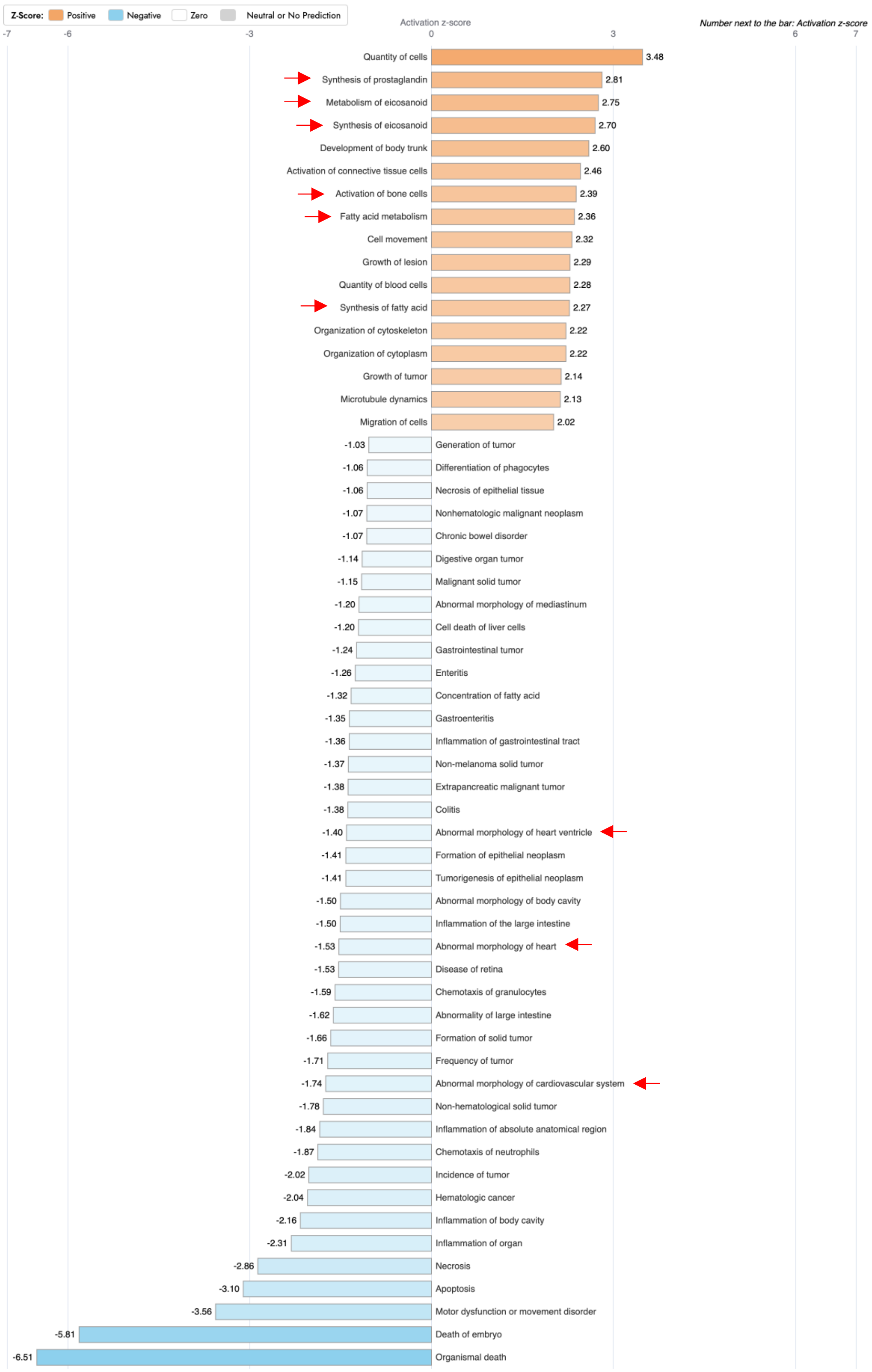

©2000 - 2025 QIAGEN. All rights reserved.

Figure S5. Disease association of differentially expressed genes in AngII-treated mice compared to control group.

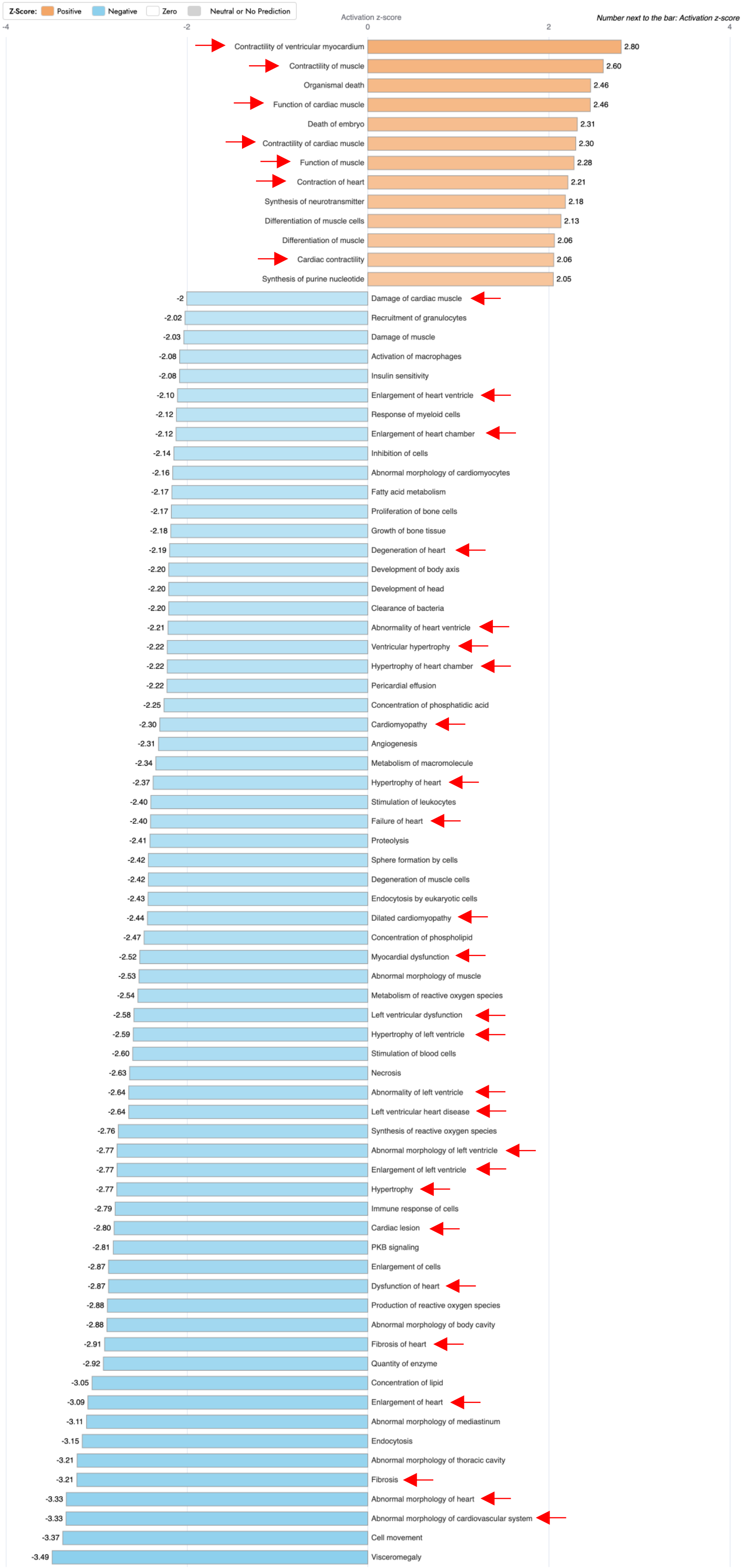

©2000 - 2025 QIAGEN. All rights reserved.

Figure S6. Disease association of differentially expressed genes in (LY+AngII)-treated group compared to AngII-treated group.

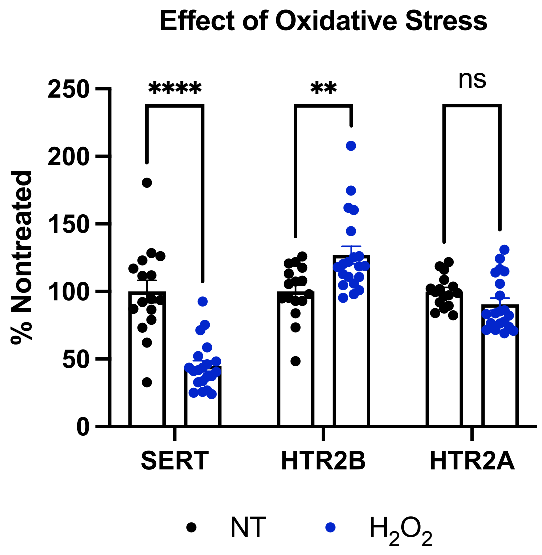

Figure S7. Effects of oxidative stress on serotonin genes in human AVICs. Acute oxidative stress induction in wild type human AVICs by 100μM H_2O_2 suppresses SERT expression and increases HTR2B expression. Data shown as mean±SEM. ** $p < 0.05$, **** $p < 0.005$ by unpaired t-tests.
