# Supplementary material for: Serotonin transporter downregulation is associated with aortic stenosis, and early profibrotic remodeling is mitigated by pharmacological inhibition of HTR2B receptor
Source: Front Cardiovasc Med. 2026 Feb 12;13:1729078. doi: 10.3389/fcvm.2026.1729078 (PMC12935953; doi:10.3389/fcvm.2026.1729078)
Supplement: Supplementary file 2 [file Table1.docx]

**SUPPLEMENTAL DATA:**

**Table S1: Complete List of Mice Echocardiographic Data Collected from NT, LY, AngII, and AngII+LY Mice**

|  | None-  Treated | LY | Ang II | AngII + LY | AngII Post Pump | AngII + LY Post Pump |
| --- | --- | --- | --- | --- | --- | --- |
| **Velocity Time Integral** | 39.40 (±9.51) | 28.12 (±7.80) | 58.77 (±11.89) | 51.79 (±26.01) | 61.86 (±6.87) | 62.10 (±17.23) |
| **Mean Velocity** | 596.22 (±55.89) | 519.68 (±148.34) | 1046.61 (±203.97) | 831.01 (±380.99) | 927.44 (±131.52) | 780.20 (±462.27) |
|  |  |  |  |  |  |  |
| **Mean Gradient** | 1.45 (±0.24) | 1.15 (±0.69) | 4.54 (±1.59) | 3.29 (±3.44) | 3.48 (±0.94) | 3.00 (±3.39) |
| **Peak Velocity** | 1020.60 (±60.17) | 856.55 (±248.36) | 1748.26 (±334.26) | 1385.02 (±657.37) | 1534.60 (±239.60) | 1272.10 (±744.61) |
|  |  |  |  |  |  |  |
| **Peak Gradient** | 4.20 (±0.50) | 3.10 (±1.84) | 11.75 (±5.52) | 9.21 (±10.48) | 9.62 (±2.87) | 7.97 (±8.79) |
|  |  |  |  |  |  |  |
| **Heart Rate** | 415.20 (±37.05) | 487.75 (±33.53) | 480.36 (±67.52) | 457.20 (±65.99) | 475.60 (±45.09) | 400.67 (±91.74) |
|  |  |  |  |  |  |  |
| **Left Ventricular Systolic Diameter** | 3.00 (±0.19) | 2.58 (±0.29) | 2.64 (±0.51) | 2.41 (±0.35) | 3.04 (±0.30) | 2.90 (±0.52) |
|  |  |  |  |  |  |  |
| **Left Ventricular Diastolic Diameter** | 3.86 (±0.18) | 3.52 (±0.15) | 3.65 (±0.37) | 3.47 (±0.42) | 3.92 (±0.28) | 4.10 (±0.20) |
|  |  |  |  |  |  |  |
| **Left Ventricular Systolic Volume** | 34.72 (±5.86) | 23.88 (±6.80) | 26.78 (±11.03) | 20.90 (±7.40) | 36.66 (±9.54) | 33.87 (±12.93) |
|  |  |  |  |  |  |  |
| **Left Ventricular Diastolic Volume** | 64.92 (±7.09) | 52.35 (±5.87) | 56.82 (±13.95) | 51.32 (±14.39) | 66.14 (±11.56) | 75.70 (±8.50) |
|  |  |  |  |  |  |  |
| **Stroke Volume** | 30.20 (±4.83) | 28.48 (±3.45) | 30.03 (±5.88) | 30.42 (±8.36) | 29.48 (±4.82) | 41.83 (±6.10) |
|  |  |  |  |  |  |  |
| **Ejection Fraction** | 46.60 (±6.15) | 54.85 (±8.25) | 54.46 (±12.08) | 59.59 (±6.56) | 45.00 (±6.93) | 56.10 (13.47) |
|  |  |  |  |  |  |  |
| **Fractional Shortening** | 22.98 (±3.66) | 28.02 (±5.19) | 28.28 (±9.16) | 28.06 (±9.59) | 22.08 (±4.11) | 29.50 (±9.21) |
|  |  |  |  |  |  |  |
| **Cardiac Output** | 12.62 (±2.81) | 13.95 (±2.57) | 14.35 (±2.94) | 13.82 (±4.14) | 14.06 (±3.03) | 16.43 (±2.20) |
|  |  |  |  |  |  |  |
| **Left Ventricular Mass** | 124.56 (±23.35) | 110.53 (±20.27) | 132.74 (±17.70) | 132.34 (±32.15) | 137.10 (±21.42) | 170.80 (±45.84) |
|  |  |  |  |  |  |  |
| **LV Anterior Wall Thickness (Sys)** | 1.30 (±0.19) | 1.18 (±0.10) | 1.45 (±0.27) | 1.39 (±0.14) | 1.30 (±0.07) | 1.50 (±0.20) |
|  |  |  |  |  |  |  |
| **LV Anterior Wall Thickness (Dia)** | 0.96 (±0.18) | 0.83 (±0.05) | 1.02 (±0.15) | 0.98 (±0.09) | 0.92 (±0.04) | 0.93 (±0.06) |
|  |  |  |  |  |  |  |
| **LV Posterior Wall Thickness (Sys)** | 0.98 (±0.08) | 1.18 (±0.13) | 1.27 (±0.33) | 1.40 (±0.24) | 1.12 (±0.24) | 1.43 (±0.25) |
|  |  |  |  |  |  |  |
| **LV Posterior Wall Thickness (Dia)** | 0.76 (±0.15) | 0.95 (±0.17) | 0.94 (±0.16) | 1.02 (±0.20) | 0.92 (±0.19) | 1.03 (±0.23) |
|  |  |  |  |  |  |  |

Numerical data in parentheses represents standard deviation for functional echocardiographic parameters.
